# RAMP: A Design Sprint for Longitudinal Curriculum Integration

**DOI:** 10.1007/s40670-026-02892-4

**Published:** 2026-09-26

**Authors:** Munder Zagaar, Claire Horner

**Affiliations:** 1 Department of Biochemistry and Molecular Pharmacology, Baylor College of Medicine, School of Medicine, Curriculum Office, One Baylor Plaza, Houston, TX 77030 USA; 2 Center for Medical Ethics and Health Policy, Baylor College of Medicine, School of Medicine, One Baylor Plaza, Houston, TX 77030 USA

**Keywords:** Faculty development, Curriculum planning, Curriculum mapping, Design sprint, Curricular threads, Longitudinal integration

## Abstract

Health professions programs increasingly seek to integrate cross-cutting curricular content across phases, yet faculty often lack shared processes for planning and implementing this work. We developed RAMP (Reflect, Anchor, Map, Plan), a curriculum design sprint that guides faculty teams through four structured steps to produce concrete integration products within a single session. RAMP was introduced in a 60-min sprint at a regional conference. Fourteen participants (*N* = 14; RR = 100%) completed a post-workshop evaluation. All reported satisfaction, and most indicated likelihood of recommending the session. Preliminary findings suggest RAMP is feasible for collaborative curriculum planning.

**Supplementary Information:**

The online version contains supplementary material available at https://doi.org/10.1007/s40670-026-02892-4.

## Background

Longitudinal integration of cross-cutting curricular content is a recognized priority in health professions education [1, 2]. Whether organized as threads, themes, strands, or pathways, this content requires coordinated decisions about scope, progression, placement, and assessment from stakeholders who often work independently [1, 2]. Curriculum committees may set broad priorities, yet faculty responsible for implementation frequently lack practical processes for defining developmental progression and aligning assessment across phases [3–5].

Existing curriculum design approaches, including backward design and constructive alignment, offer important conceptual frameworks but do not provide a stepwise implementation workflow for the practical design work that integration requires. Backward design begins by identifying desired learning outcomes before instructional and assessment strategies are selected, whereas constructive alignment emphasizes explicit alignment among learning objectives, educational activities, and assessment methods [6–8]. Design sprint methodologies, which compress iterative work into structured, time-boxed sessions that produce tangible deliverables, have been applied in product development and course design but have not been adapted for the collaborative work of longitudinal curriculum integration [9, 10]. Without structured support, integration efforts may remain implicit, fragmented, or dependent on informal coordination [1, 3, 5, 11, 12].

To address this gap, two curriculum leaders developed RAMP (Reflect, Anchor, Map, Plan), a curriculum design sprint that guides faculty teams to produce concrete integration products within a single session. RAMP adapts sprint principles by compressing what typically requires months of committee deliberation into a structured, time-boxed collaborative process with defined deliverables at each phase [9]. The framework operationalizes principles from constructive alignment, backward design, and reflective practice into a repeatable workflow tailored for integrating longitudinal curricular content [6–8]. The objectives of this study were to (1) describe the RAMP framework and its theoretical foundations, (2) evaluate its feasibility and participant perceptions during an initial conference workshop implementation, and (3) characterize the curriculum integration products generated through the process.

## Activity

RAMP was delivered as a 60-min curriculum design sprint at a regional health professions education conference. The session targeted medical educators, course and thread directors, and curriculum administrators involved in longitudinal integration of cross-cutting curricular content. A curricular thread was defined as a knowledge domain, skill set, or disciplinary area developed progressively across courses or curricular phases. In this implementation, we used curricular content for two sample threads, pharmacology and ethics.

**Fig. 1:**
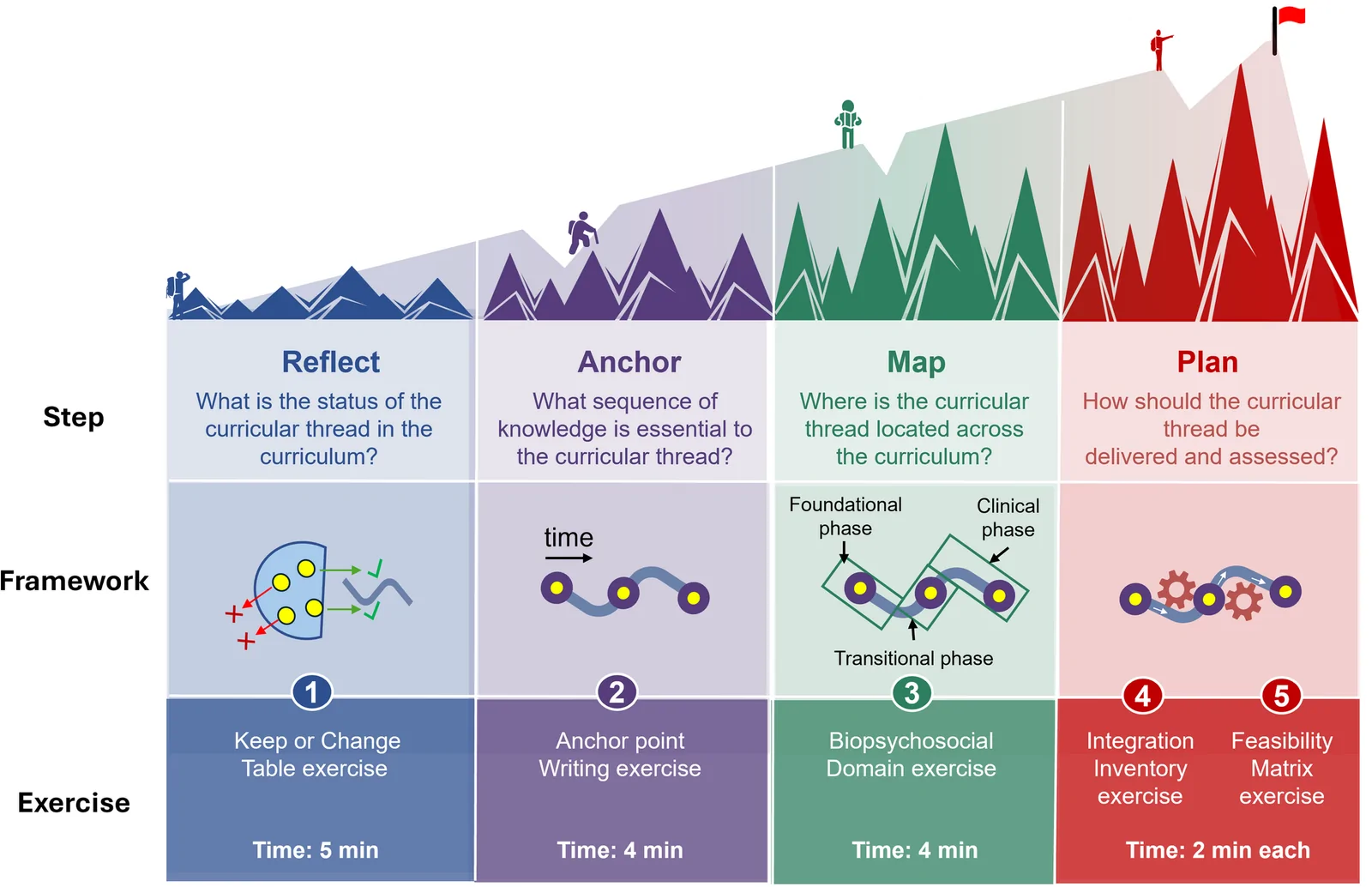
The RAMP design sprint framework for longitudinal curriculum integration. RAMP is a structured, time-boxed process organized into four sequential steps: Reflect, Anchor, Map, and Plan. Reflect uses a keep-or-change table to summarize current content coverage; Anchor uses sequenced anchor points to define essential knowledge and skills over time; Map uses a two-dimensional grid to visualize depth (vertical axis) and breadth across biopsychosocial domains (horizontal axis); and Plan uses a thread taxonomy inventory and impact-feasibility matrix to classify the content and prioritize instructional strategies

The sprint consists of four steps, each introduced with a concise explanation followed by a guided exercise (Fig. 1; see Online Resource 1 for examples). In Reflect, participants reviewed historical curricular materials and evaluation data to identify strengths, gaps, and priorities related to a curricular thread. This phase operationalizes reflective practice by prompting faculty to examine assumptions and identify mismatches between intended and implemented curricula before redesign begins [3, 11]. In Anchor, participants translated observations into a small set of progressive, outcome-aligned anchor points articulating what learners should know or be able to do at key developmental stages. For this workshop, the anchor points represented foundational, transitional, and clinical-level expectations. This phase applies backward design logic by beginning with desired outcomes and working backward to define expectations [7, 8]. In Map, participants placed anchors onto a two-dimensional curriculum grid to identify where learning and assessment opportunities existed or were absent. The vertical axis represented the depth of coverage across the three anchor points, while the horizontal axis represented the breadth across Engel’s biopsychosocial domains: biological, individual, and social/population [13]. This structure made constructive alignment decisions explicit and visible across both dimensions [6]. In Plan, participants classified their thread as independent (greater control over instruction and assessment, less integration) or dependent (less control, more integration) using a scoring inventory, then identified feasible teaching and assessment strategies using an impact-feasibility matrix, along with responsible stakeholders and governance pathways [14].

Sprint materials were intentionally low-cost, including pre-printed worksheets, sticky notes, laminated curriculum grids, and dry-erase tools as shown in Online resource 1 (SM 1). Participants worked in five small groups using two sample thread scenarios: pharmacology, representing a dependent thread, and ethics, representing an independent thread. Each scenario included a legacy course status card summarizing learning objectives, faculty composition, assessment methods, and prior student evaluation data. A content-neutral example was used to model each exercise before participants applied the process to their assigned scenario. At the conclusion of the session, participants completed an anonymous post-workshop survey with Likert-style items on satisfaction, most useful steps, and perceived next steps, corresponding to Kirkpatrick levels 1 and 2 [15]. Quantitative responses were summarized descriptively. Two authors independently reviewed open-ended comments, generated preliminary codes, and resolved coding differences through discussion before organizing responses into thematic categories for formative feedback. During manuscript preparation, Microsoft 365 Copilot Chat GPT 5.5 was used for limited editorial assistance and preliminary analysis; all outputs were reviewed, verified, and revised by the authors. The study was approved by the Institutional Review Board at [institution blinded for review] (H-56377).

## Results and Discussion

Each group completed structured deliverables across all four RAMP steps during the sprint. These deliverables included prioritized gap analyses, developmental anchors, visual maps showing thread coverage across three curriculum phases, and implementation plans outlining near-term strategies and integration approaches. Representative outputs included developmental anchors for learner progression, curriculum maps illustrating thread placement across phases, and implementation plans identifying strategies, stakeholders, and governance considerations.

All fourteen participants (*N* = 14; RR = 100%) completed the post-workshop evaluation. Ratings of satisfaction, likelihood of recommending, and perceived usefulness of materials were uniformly positive and are summarized in Table 1.

**Table 1:** Sprint evaluation results (*N* = 14)

Item	Response	*n*	%
Overall Satisfaction	Very Satisfied	11	78.6
	Satisfied	3	21.4
	Neutral	0	0.0
	Dissatisfied	0	0.0
Likelihood of Recommending	Very Likely	8	57.1
	Likely	4	28.6
	Neutral	1	7.1
	Unlikely	1	7.1
Materials Useful^a^	Strongly Agree	9	64.3
	Agree	3	21.4
	Neutral	2	14.3

^a^The Materials Useful item used a five-point agreement scale. No participants selected "Disagree" or "Strongly Disagree"

Reflect was most frequently identified as the most valuable phase (7/13, 53.8%), followed by Map (4/13, 30.8%). Qualitative feedback highlighted the practical applicability of the framework, with participants describing the workshop as useful for guiding integration planning (Table 2).

**Table 2:** Qualitative themes from open-ended feedback

Theme	Representative quote(s)	*n*
Practical Applicability	*“Really well thought out workshop that every curriculum team would benefit from attending.”* *“Very informative and helpful in explaining threading through curriculum.”*	4
Instructional Design Balance	*“Nice combo of lecture and interactivity.”* *“The session was just almost planned too well around an exercise—leave more time for participant sharing of best practices.”*	2
Framework Clarity Needs	*“I would have liked to know more about how you generated this framework and more about how to do the individual steps.”*	2
Terminology Refinement	*“I think ‘designing threads…’ would be a more apt title. The word ‘managing’ gives more a sense of what to do after the thread is established/created.”*	1

*n* refers to coded segments; a single comment may contribute to multiple themes. Eight of 14 participants provided open-ended comments

Four qualitative themes emerged from open-ended feedback (Table 2). Practical applicability was the most frequently cited theme, with participants describing the sprint as useful for guiding integration planning. Instructional design balance and framework clarity needs reflected a desire for more time for application and deeper explanation of the framework’s origins. One participant recommended reframing the workshop title to emphasize thread design rather than management. Collectively, these themes reinforced the quantitative findings while identifying actionable targets for future iterations.

These findings suggest that RAMP is feasible as a curriculum design sprint and that faculty teams can produce usable planning products within a single structured session. The primary contribution of this work is the adaptation of sprint methodology for curriculum integration, providing a shared, repeatable process that compresses distributed committee work into a time-boxed collaborative format with concrete deliverables [4, 9].

RAMP introduces features not typically addressed by general curriculum design models. The Map phase uses a biopsychosocial lens to define content breadth across biological, individual, and social/population domains, moving beyond simple temporal placement to visualize both depth and contextual scope [13]. The Plan phase introduces a thread taxonomy that classifies content as independent or dependent based on dedicated time requirements, interdependence with other threads, and specialized faculty needs, with implications for governance and assessment control [14]. Unlike broad program-level models such as the six-step approach or SPICES (Student-centered, Problem-based, Integrated, Community-based, Elective, and Systematic), RAMP operates at the thread level, targeting the specific collaborative decisions that faculty teams face when translating integration goals into coordinated action [16, 17].

The finding that Reflect was perceived as the most valuable phase aligns with perspectives that view structured reflection as a prerequisite for meaningful redesign rather than an incidental step [11, 18]. This is consistent with sprint methodology, in which the initial framing phase is often identified as the most critical for aligning team understanding before solution generation begins [9].

RAMP may be particularly useful in settings where responsibility for longitudinal integration is distributed across faculty, where integration efforts have stalled, or where time for curriculum planning is limited. The sprint format is well suited to these constraints because it protects dedicated time for collaborative design work and produces tangible outputs that can inform subsequent governance decisions.

Several limitations warrant consideration. The sample was small (N = 14) and drawn from a single regional conference, which limits generalizability and introduces the possibility of selection bias. Participants were self-selected attendees with an existing interest in curriculum innovation, which may have contributed to the favorable perceptions reported. However, the 100% response rate and triangulation of quantitative and qualitative data strengthen the interpretability of these preliminary findings. The evaluation captured participant reactions and perceived usefulness (Kirkpatrick levels 1 and 2) but did not include follow-up assessment of behavior change, implementation fidelity, or curricular outcomes (levels 3 and 4) [15]. This is consistent with early dissemination of faculty development resources, and to support transferability, we provide complete, implementation-ready materials in Online resource 1. The 60-min format constrained the depth of engagement with each phase; participant feedback confirmed that additional time for application and peer sharing would strengthen future iterations. Participants worked with exemplar scenarios rather than local curricular data, which supported feasibility testing but may have limited the perceived authenticity of the exercises.

Future work should examine use of RAMP with institutional teams addressing active curricular priorities using local curricular data and evaluate implementation fidelity, behavior change, and curricular outcomes corresponding to higher Kirkpatrick evaluation levels. Expanding the sprint format to allow deeper engagement, more time for peer sharing, and inclusion of case examples from multiple institutions would help demonstrate adaptability across diverse contexts. Developing pre-sprint materials that introduce the origins and rationale of the RAMP framework would allow in-session time to focus on application. Testing the sprint in other health professions programs would help clarify transferability beyond undergraduate medical education.

## Supplementary Information

Below is the link to the electronic supplementary material.

Supplementary file1 (PDF 2890 KB)

## Data Availability

The datasets generated and analyzed during the current study are available from the corresponding author on reasonable request.
