# Supplementary material for: RAMP: A Design Sprint for Longitudinal Curriculum Integration
Source: Med Sci Educ. 2026 Sep 26;36(5):2129–33. doi: 10.1007/s40670-026-02892-4 (PMC13723595; doi:10.1007/s40670-026-02892-4)

Coming Together at the Seams

Managing Threads in a Modern Integrated-Medical School Curriculum

Examples are provided for demonstration purposes only and do not represent recommended scope, sequencing, or assessment standards.

Workshop goal: Transition a subject-based course into a curricular thread using the RAMP framework.

- **Subject-based courses** allow more control over **content** and **context** but hinder intuitive integration.
- Subject-based courses can be reframed as **longitudinal threads** to enhance inter-disciplinary integration.
- Threads may be broad **super threads** (i.e. clinical skills, professional development, basic sciences, and public health)…
  - Less granular topic coverage encourages subject-based course structure, and many faculty needed to cover broad domains
- ...or more specific **sub-threads** (i.e. genetics, anatomy, physiology, oncology, pharmacology, biochemistry, ethics, clinical skills, microbiology, etc.)
  - Allows for more control and accountability, more intentional topical threading, and consistency in delivery
- **Thread directors** facilitate the coordination of subject-based content across various timeframes and settings.

The RAMP framework guides thread development

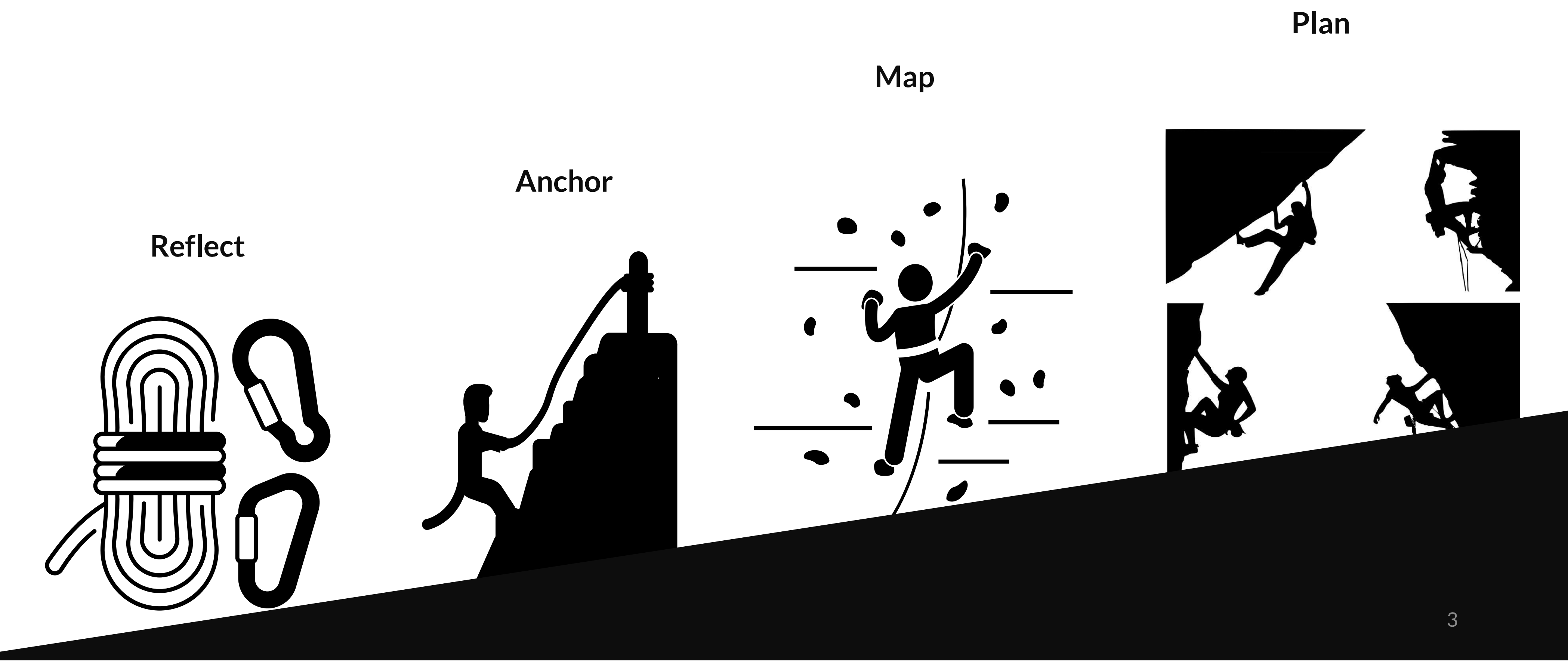

REFLECT on your thread…

Start with the end in mind – what is worth understanding?

Assess the status of your thread**.**

Reflect on **assessment and instructional methods** of your thread.

Exercise 1: What to keep, what to leave?

In this exercise, the goal is to review your past course to evaluate the strengths and weaknesses of the legacy curriculum and identify elements to keep and opportunities for improvement.

Step 1: On your own, review the "stat card" for your chosen course. Think about what worked and what didn't.

Step 2: Using sticky notes, identify aspects of the course you want to keep and elements you want to change in the new thread.

Step 3: Using the dot stickers, vote on 2 components to keep and 2 components to change. Discuss the results with your group.

Frank, Snell et al, *Med Teacher*, 2010
Joyce & Swanberg, *AMERE*, 2017
Wiggins & McTighe, 2005

Superiority of Cats Course Stat Card

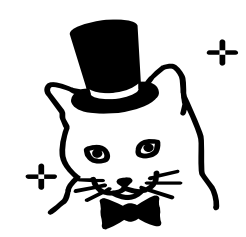

Course Duration: 10 hours
Objective: Identify why cats are top household pets
Availability: Anytime elective
Faculty: 1 clinician
Assessment: Final PowerPoint presentation
Teaching Methods:
- 7 live lectures
- 3 hands-on cat sessions

Feedback:
- **Course Rating:** High (6.8/7)
- **Social Skills:** Needs improvement in diverse pet knowledge
- **National Exam**: Scores below average over 5 years

Keep	Change
Faculty is popular	Content is not broad enough – include information about other pets
Cat encounter sessions	Address USMLE readiness

ANCHOR your thread

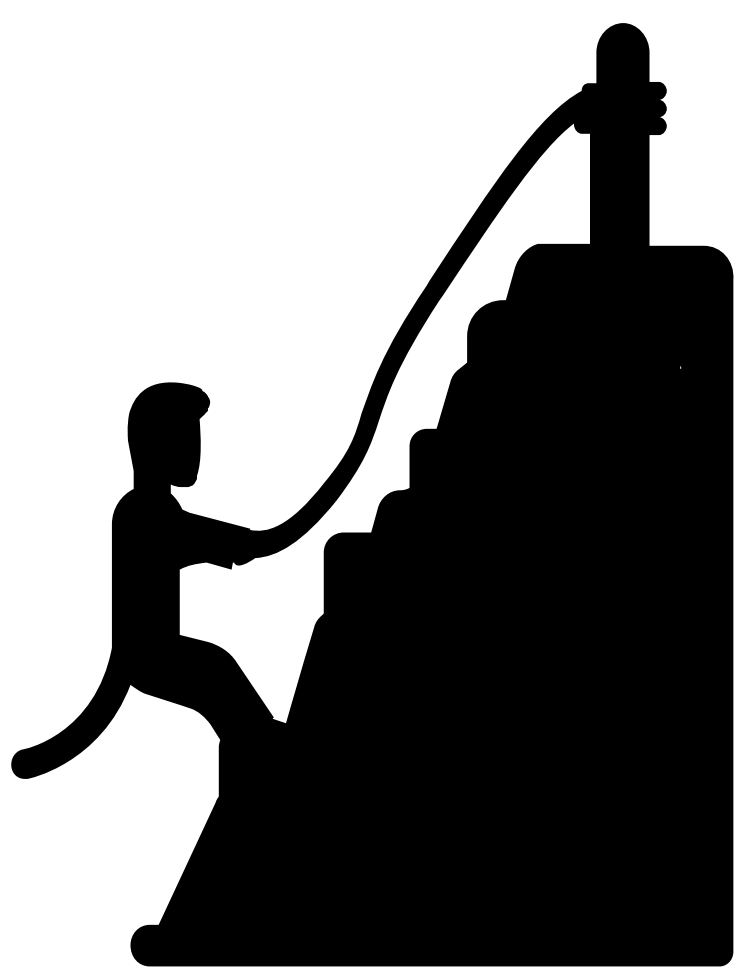

Exercise 2: Identifying major thread milestones

Imagine that a learner has gone through your ideal curriculum: what knowledge and skill sets should they have? Working backward, think about the major knowledge and skill domains that will help them get there. Generally, they will follow a logical progression:

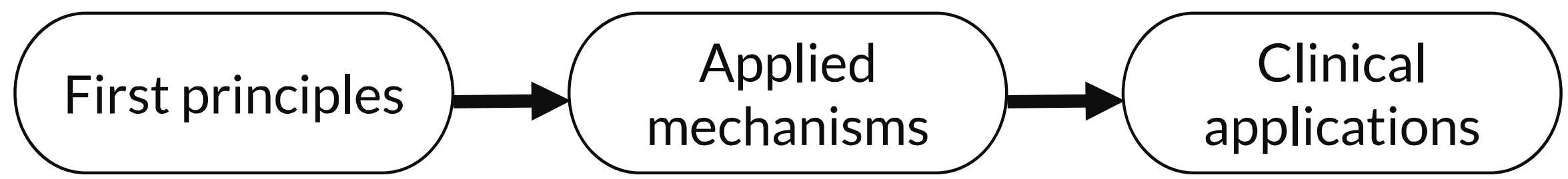

These are your anchor points. Anchor points help us understand the depth of our curriculum, while giving us a path to follow as we thread our content throughout the 4 years.

Step 1: On your own, brainstorm what you think would be 3 key skills for your thread. Or, you can use the pre-written anchor points provided on the table.

Step 2: Using the essential knowledge and skills that either you developed or we provided, sort them where you think they fit as anchor points. Discuss with your group.

Objective:
Arrange key thread skills into 3 anchor points

Phase 1. (5 min)
Work alone. Arrange the skills under their respective anchor points

Phase 2.
Participants vote on best anchors for each phase of curriculum

3 key knowledge or skill sets

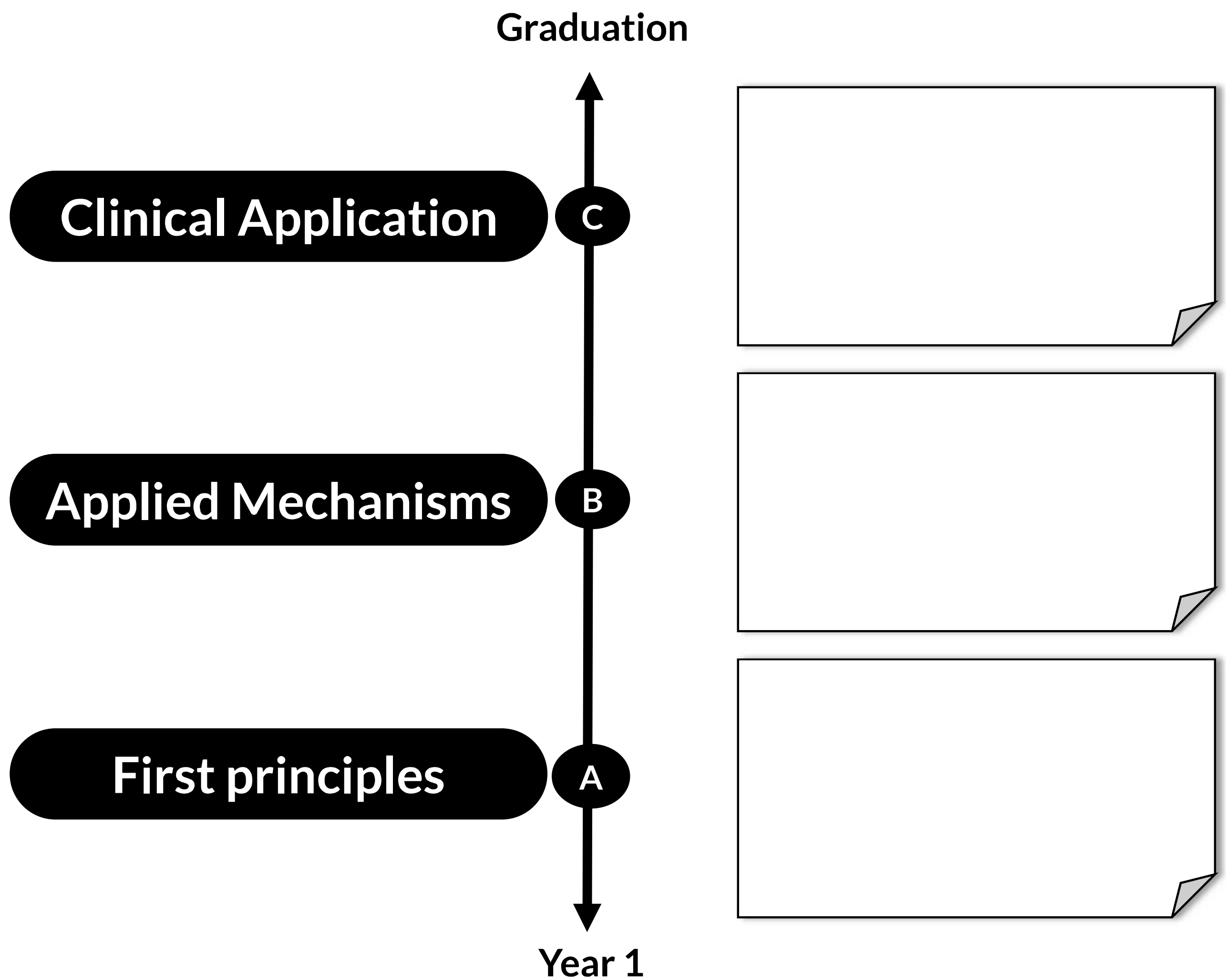

MAP
your thread

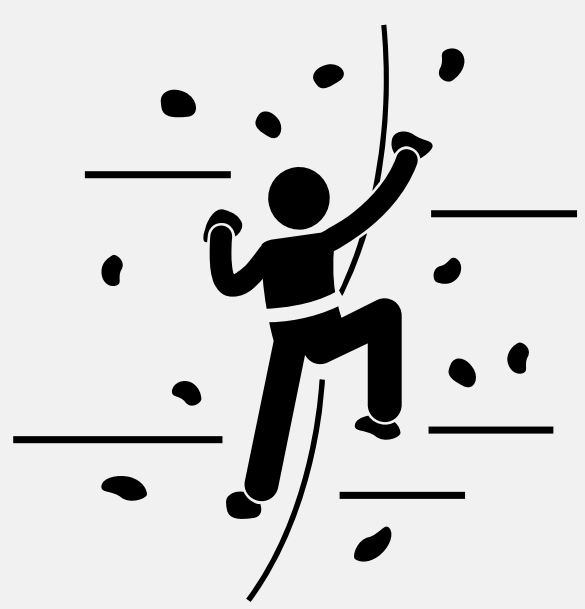

Exercise 3: Where should your anchor points go in the integrated curriculum?

In this exercise, you will do two things:

1. Think about two key dimensions of your curriculum:
   - The **depth** of your thread coverage (using anchor points)
     - How deeply are the students learning the concept?
   - The **breadth** of your thread coverage (using biopsychosocial approach)

2. Decide where in the curriculum (which courses) the anchor point concepts will be covered
   - Foundations of Medicine
   - Organ systems and clinical skills
   - Clerkships

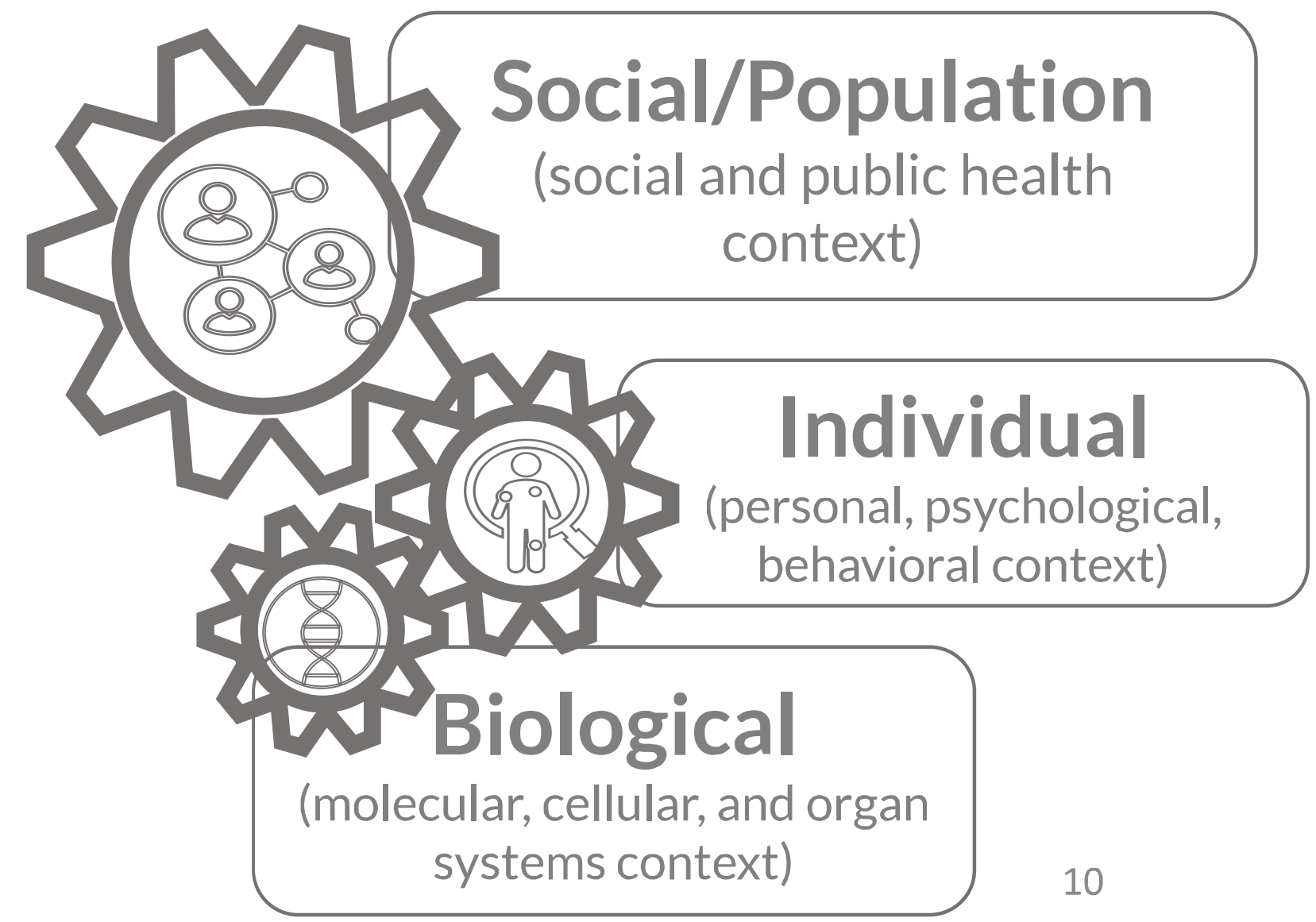

The goal of this step is to start planning where you will put your thread content in the longitudinal curriculum, using your anchor points as guides.

Adler RH. J Psychosom Res. 2009
Borrell-Carrio et al., Ann Fam Med 2004

MAP
your thread

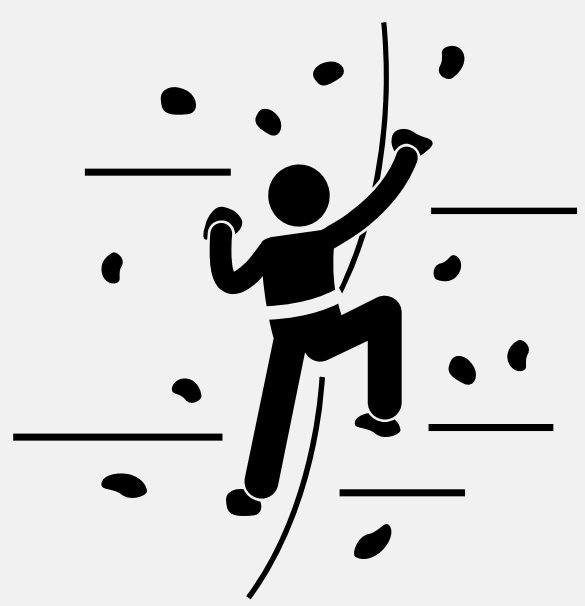

The goal of this step is to start planning where you will put your thread content in the longitudinal curriculum, using your anchor points as guides.

Exercise 3: Where should your anchor points go in the integrated curriculum?

Step 1: Place your **anchor points** (identified in exercise 2) along the y-axis.

Step 2: Using the 3 provided courses (Foundations of Medicine; Organ systems & clinical skills; and Clerkships), illustrate where in the curriculum you will cover the concepts in each anchor point (y-axis) and the scope of the content based on the biopsychosocial levels (x-axis). You can change the size and shape of the courses as needed to illustrate curriculum coverage.

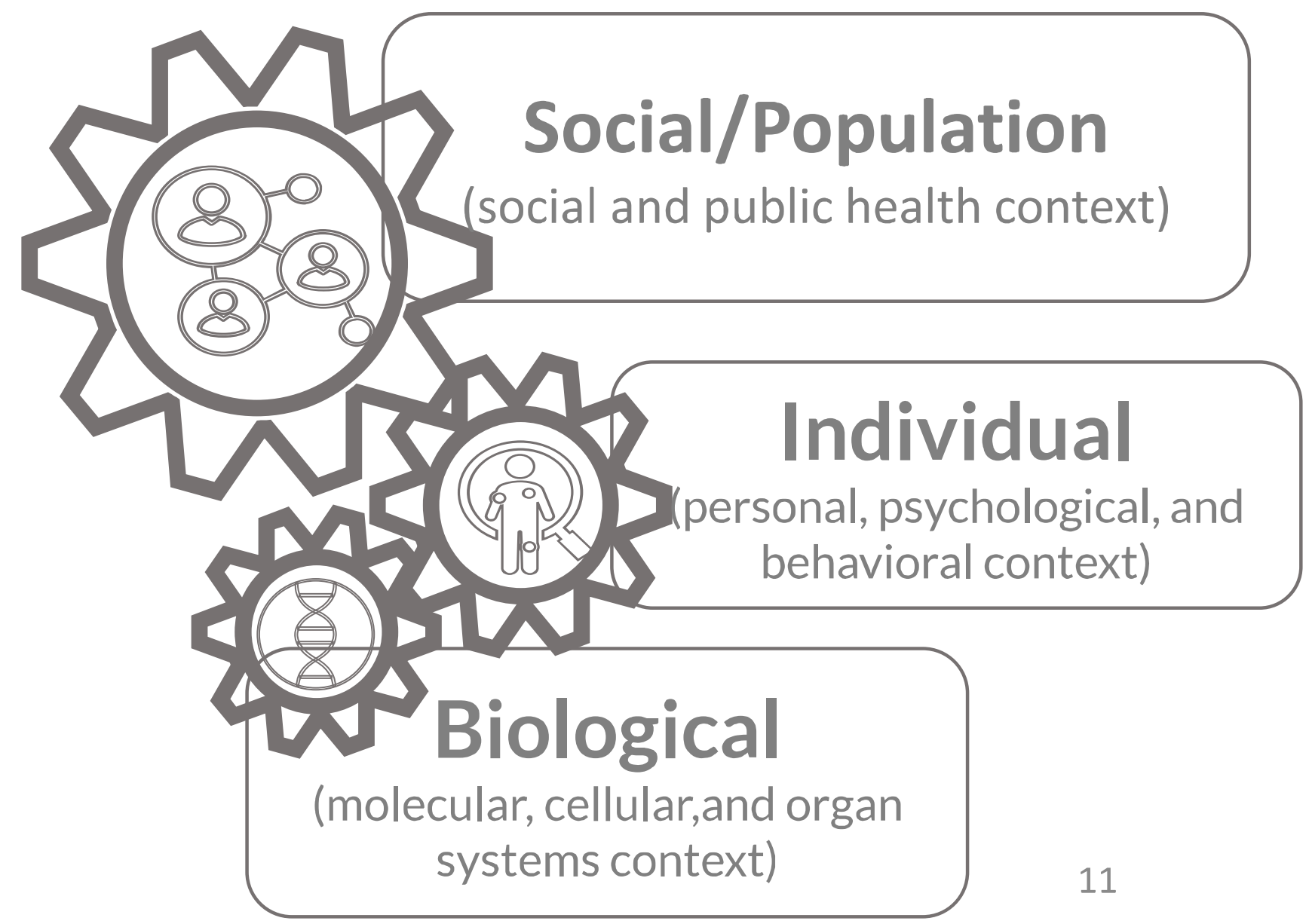

Depth and Breadth of thread

Objective:
Map depth and breadth of thread using anchor points.

Phase 1. (5 min) Move your anchor points from previous step on y-axis.
Use sample curriculum blocks below to illustrate where anchor points are covered across curriculum. They can be resized as needed.

Foundations of Medicine

Organ systems and Clinical skills

Clerkships

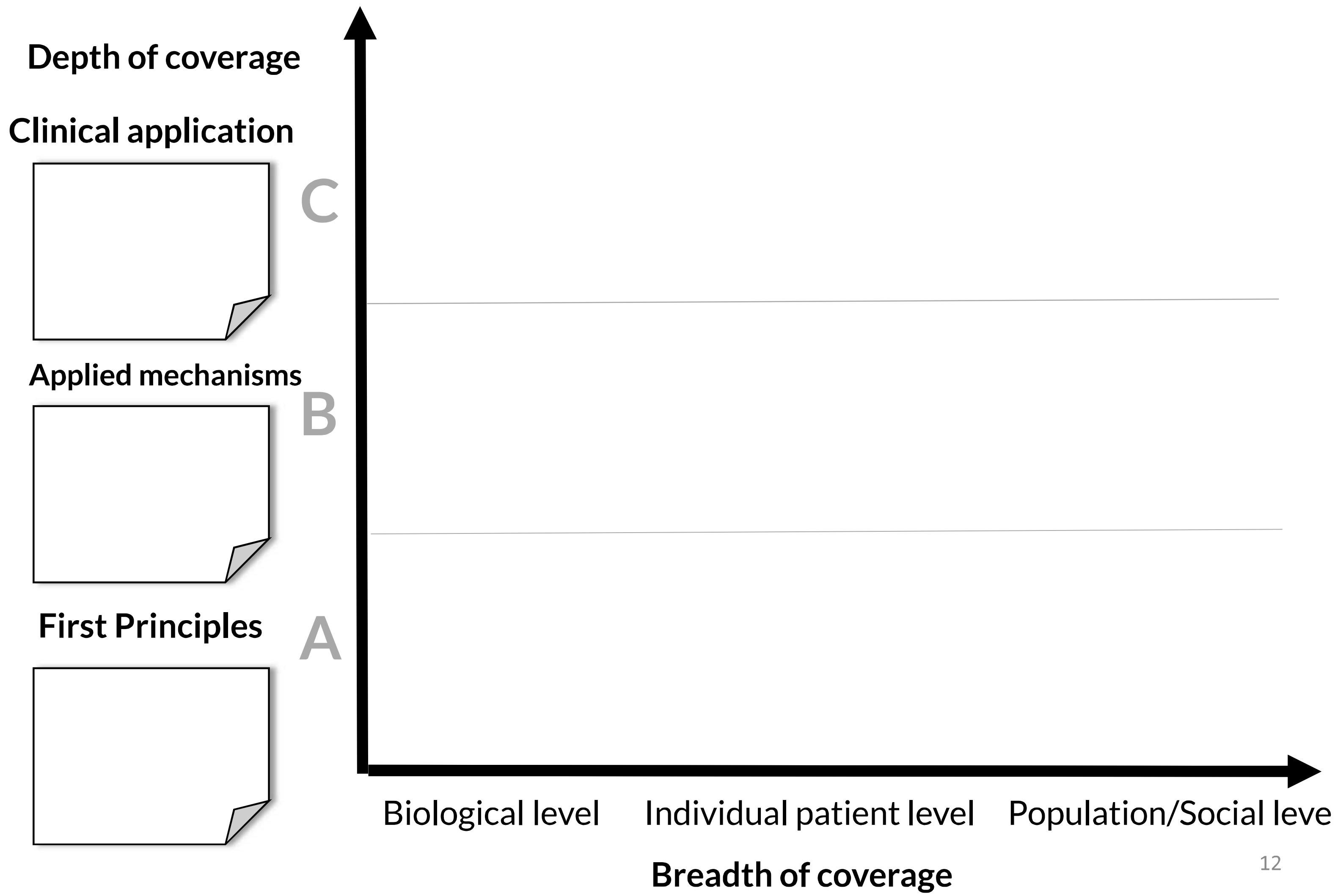

Objective:
Map depth and breadth of thread using anchor points.

Example map of Pharmacology Thread anchor points

Depth and Breadth of thread of Pharmacology

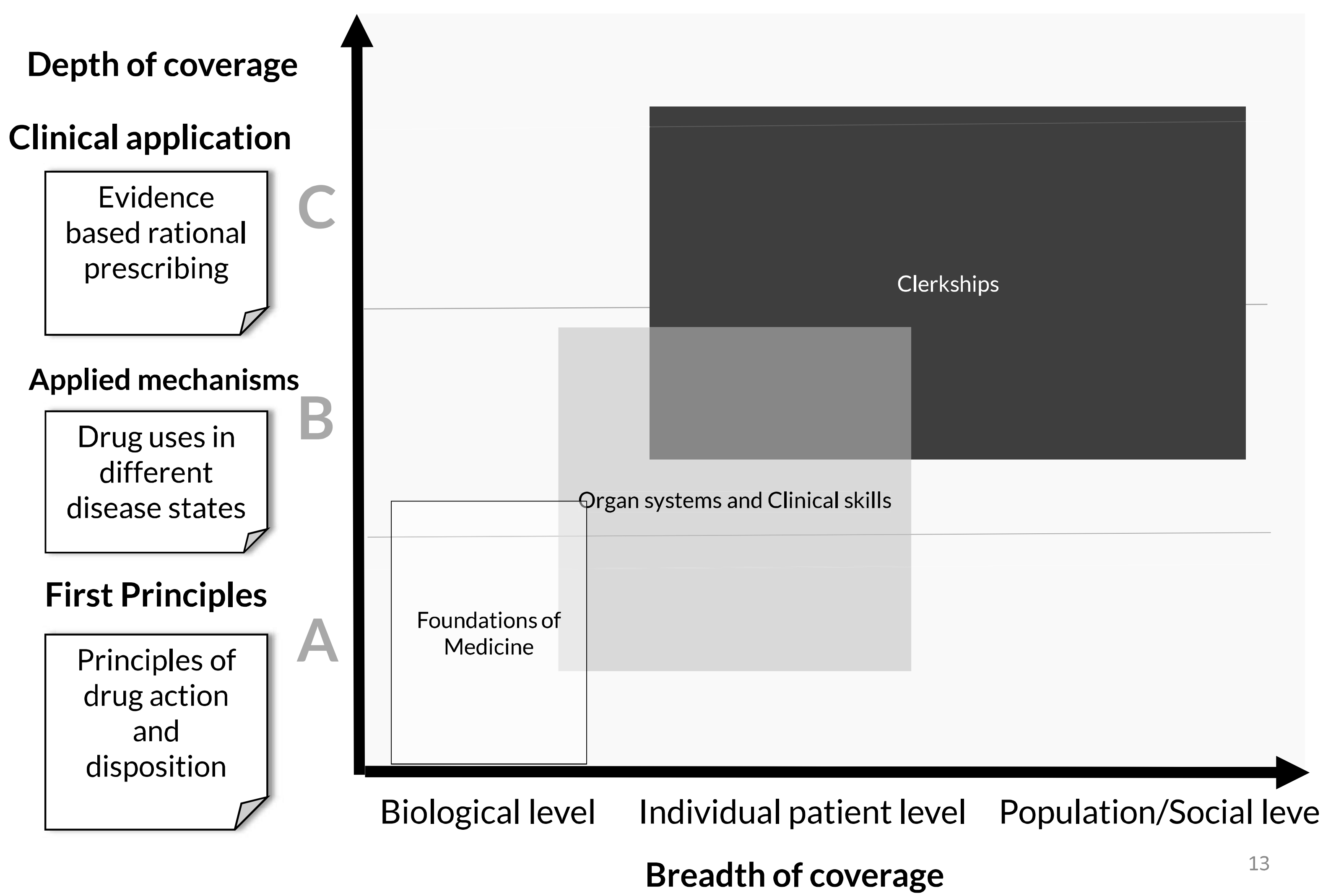

Objective:
Map depth and breadth of thread using anchor points.

Example map of Ethics Thread anchor points

Depth and Breadth of thread of Ethics

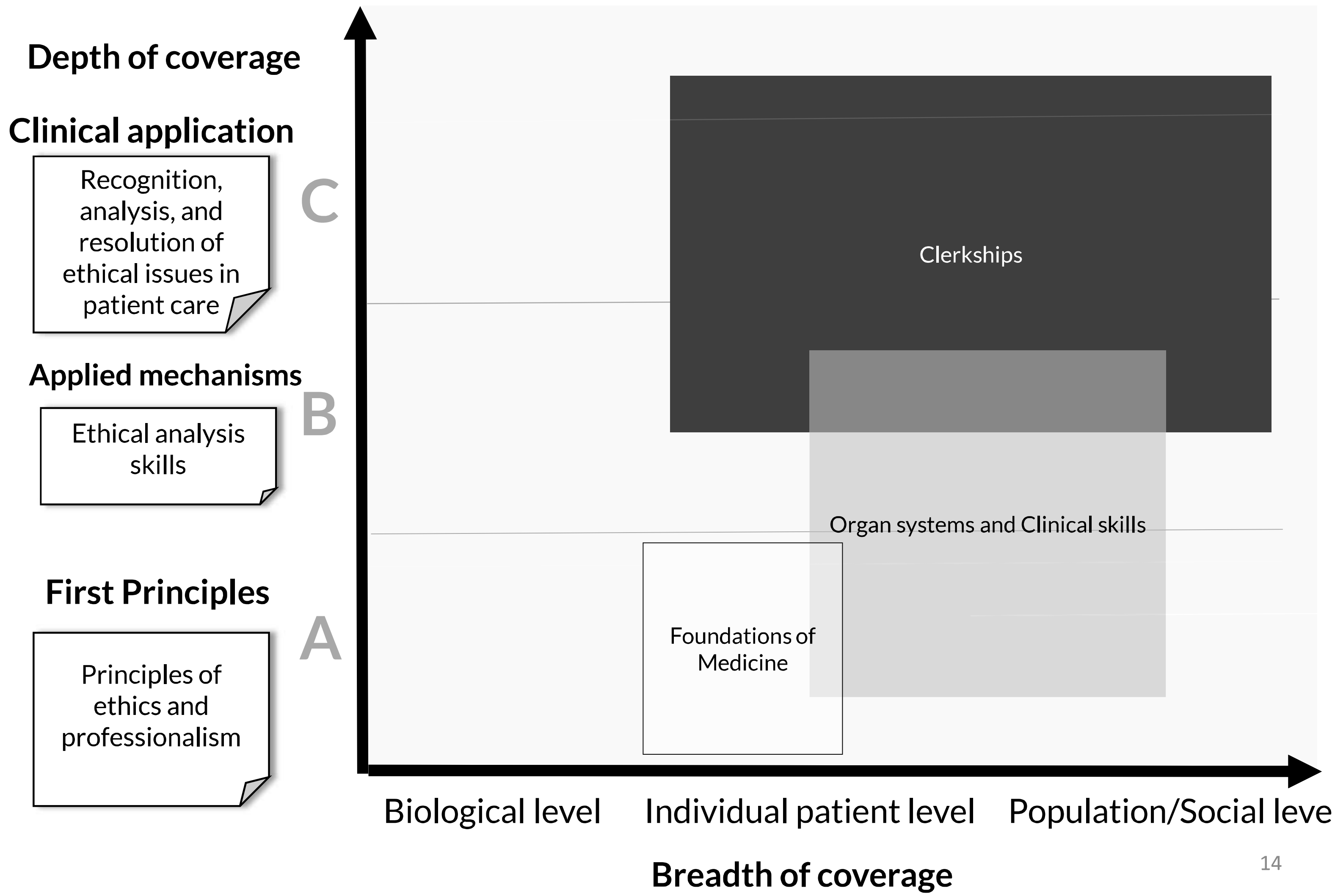

PLAN your thread

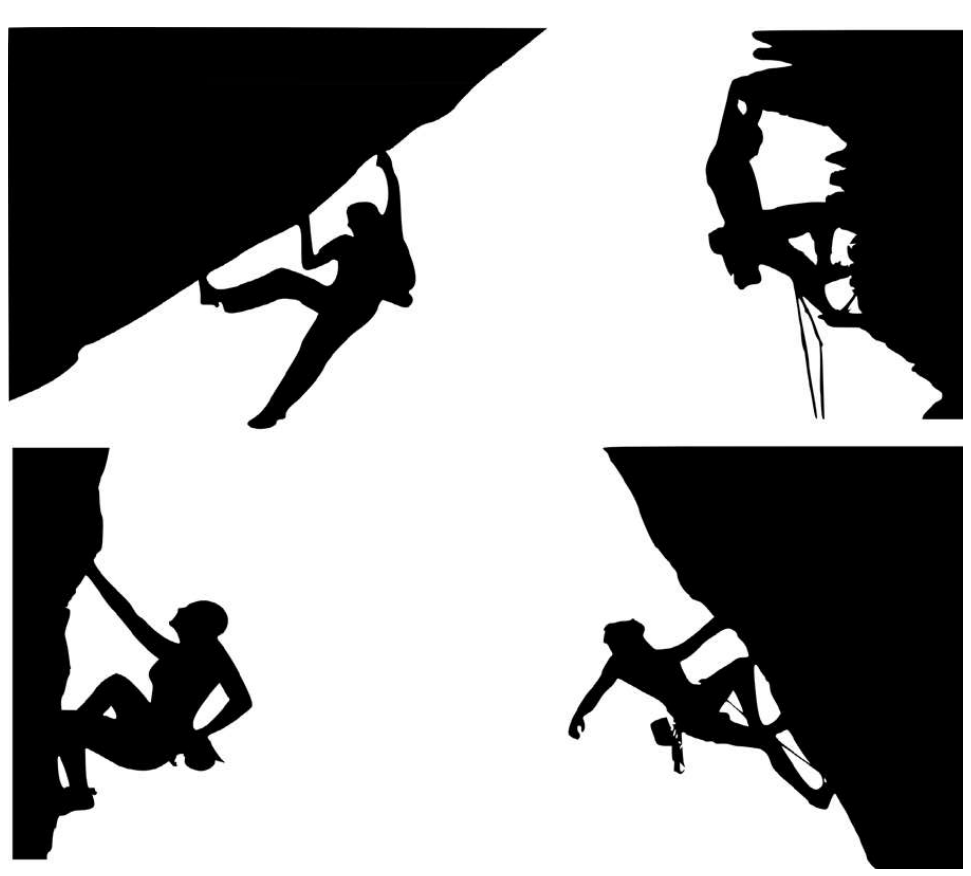

Exercise 4: Dependent or Independent?

The goal of this exercise is to determine whether your thread is a **dependent** or an **independent** thread, which will inform your approach to assessments, learning methods, and administrative organization of your thread.

Independent threads give thread directors greater control over assessment and instructional methods, but will have less integration and interdependency with other threads.

Dependent threads give thread directors less control over assessment and instructional methods, but will have more integration and interdependency with other threads.

	If yes, then:
Do >50% of thread topics require dedicated course time to cover?	+2
Is >50% of your thread interdependent on other threads?	-2
Do your thread topics require specialized thread topics to teach?	+1
Do your thread topics require special dedicated institutional resource or setting?	+1

> 2: Independent
< 2: Dependent

Score: ________

PLAN your thread

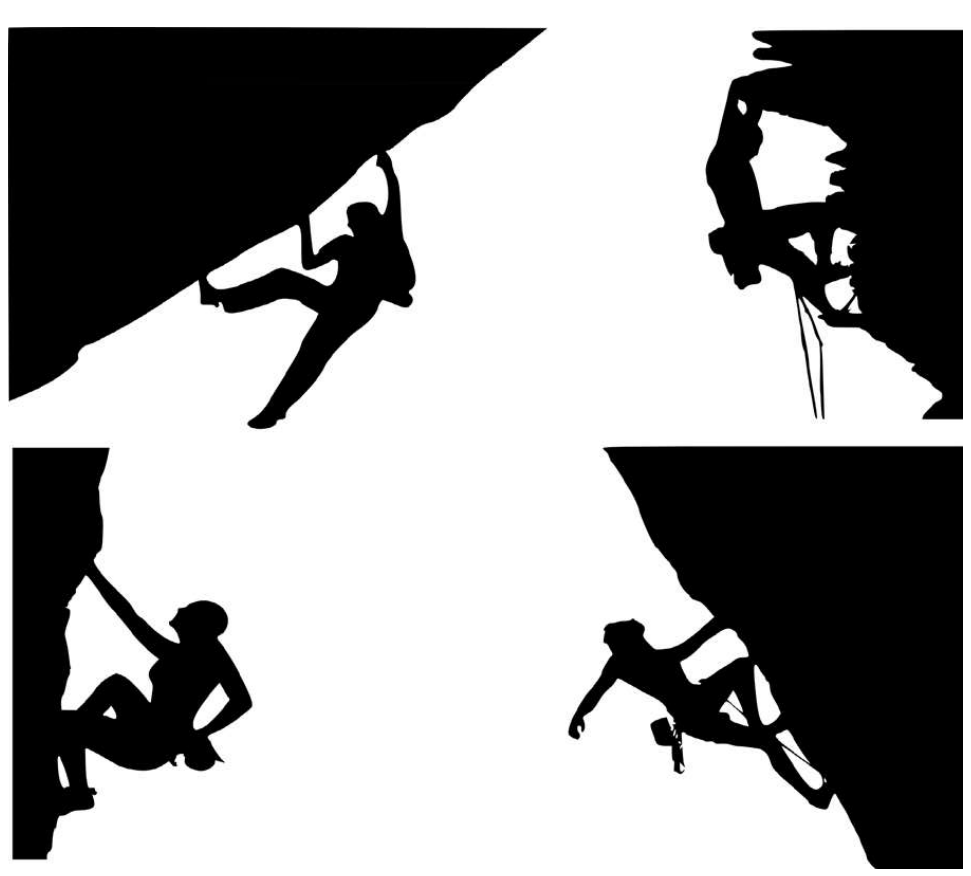

Exercise 5: How to teach & how to test

Now that you know whether your thread is independent or dependent, the goal of this exercise is to identify the **assessment** and **instructional methods** that will be most effective for your thread. Consider the following:

- What worked well and not so well in the legacy curriculum? (exercise 1)
- Are different methods needed for different anchor points? (exercise 2)
- What methods are feasible during each course? (exercise 3)
- How much control do you have over choosing the assessment methods and pedagogical approaches? (exercise 4)

Step 1: Work alone: using only anchor point A that you identified in exercise 2, identify the assessment and instructional methods that would work best for your thread content for that anchor point.

Step 2: Discuss with your group to find consensus.

Assessments	Instructional Methods

Objective:
Plan your thread delivery

Step 1. Work alone. (5 min) Using anchor point A, identify the best assessment and pedagogical strategies for your thread.

Step 2. (5 min) Participants come to consensus

How might we prioritize delivery of **Anchor Point A?**

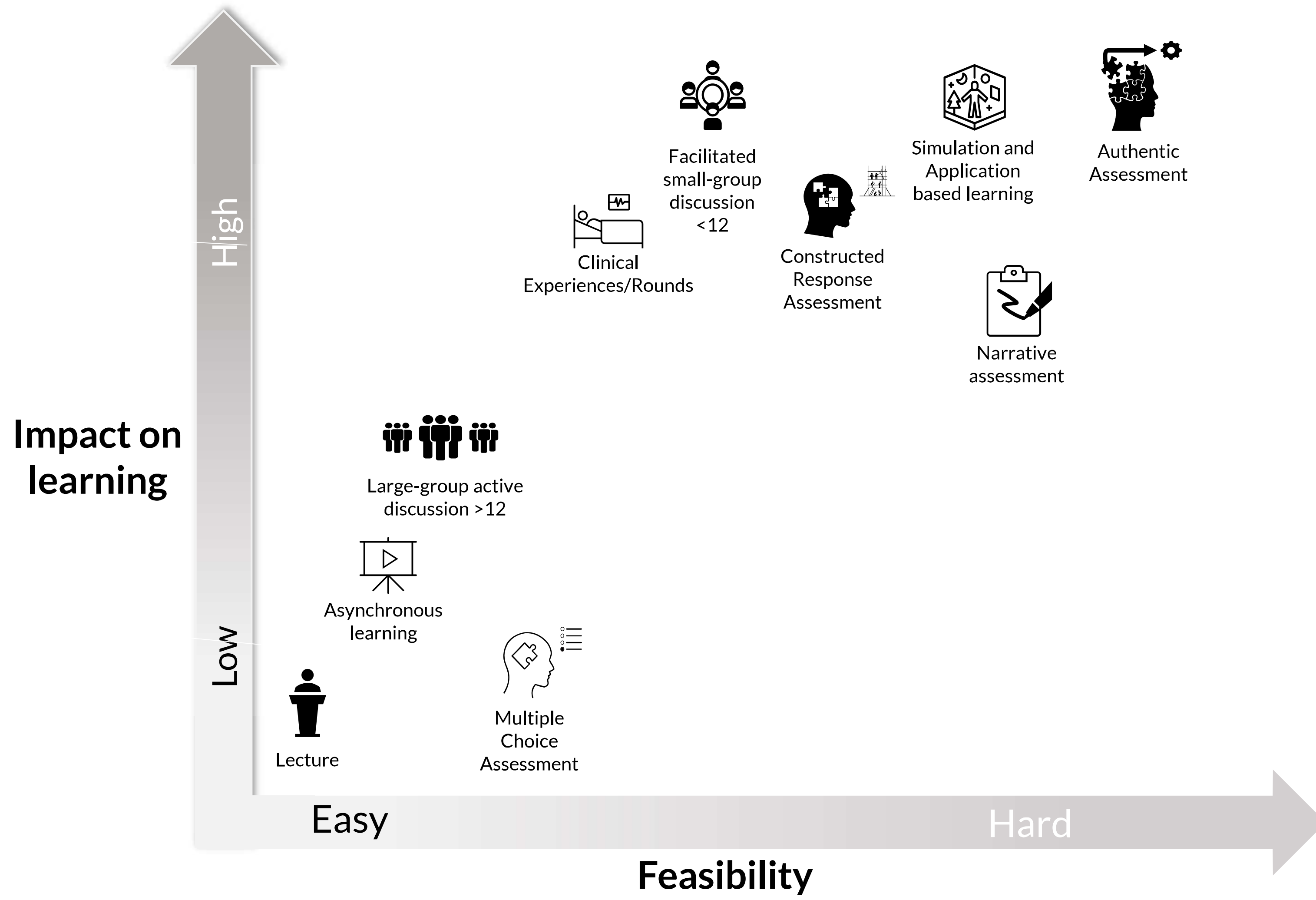

AAMC Curriculum Inventory (CI) (2012-2022)

How might we prioritize delivery of an **independent** ethics thread?

Illustration of assessment and instruction methods that are more effective and less effective for **independent** thread delivery

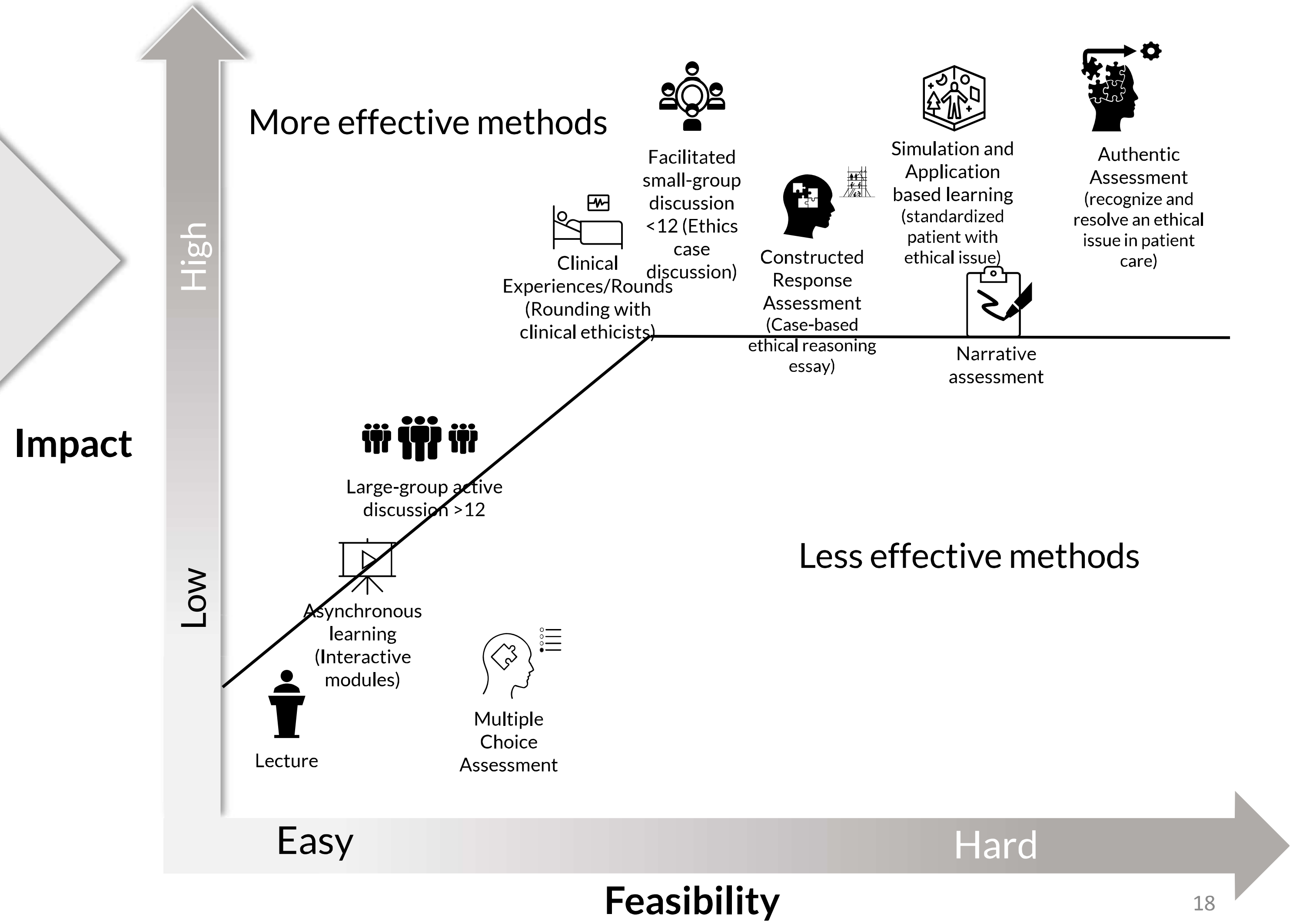

How might we prioritize delivery of a **dependent** pharmacology thread?

Illustration of assessment and instruction methods that are more effective and less effective for **dependent** thread delivery

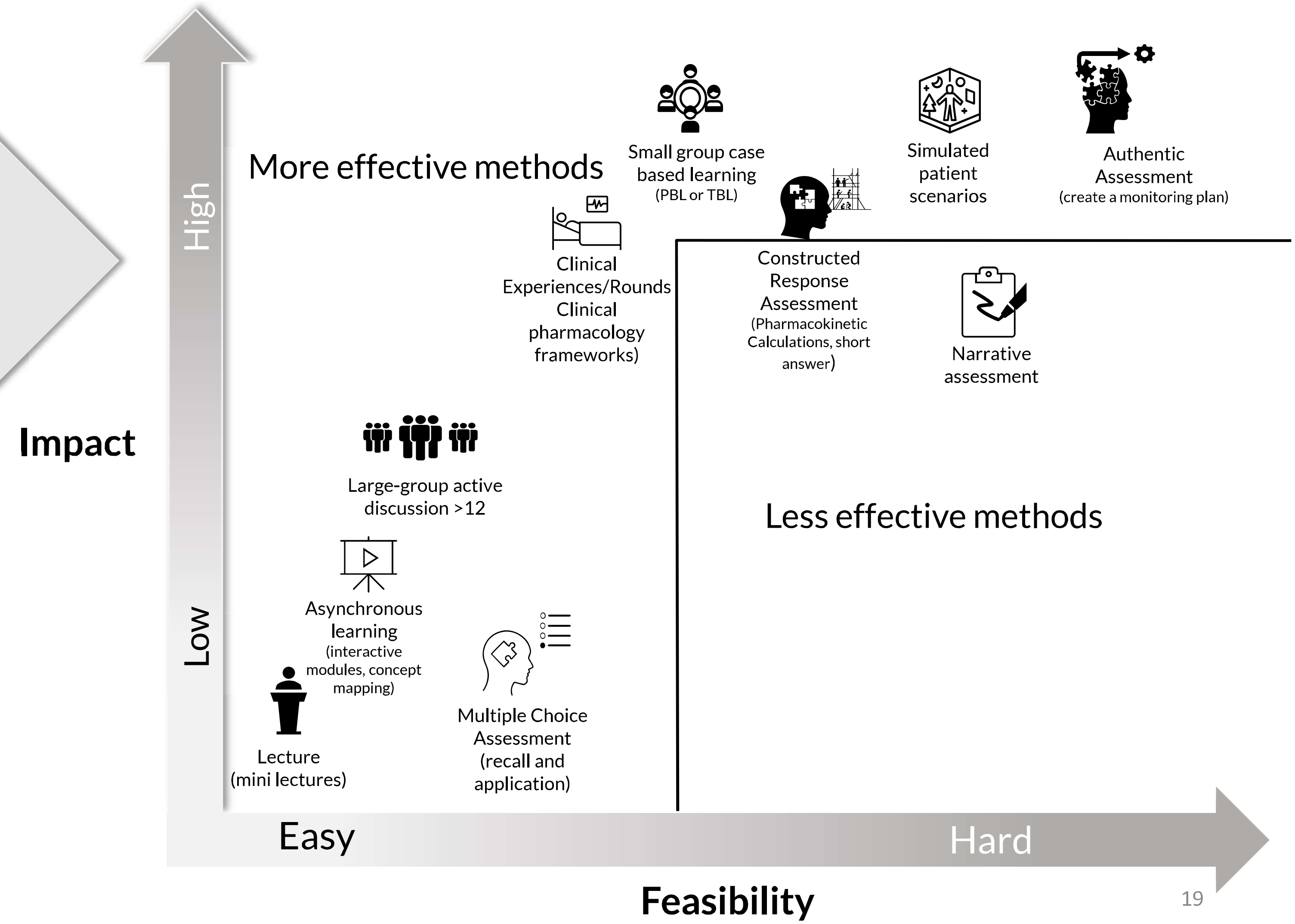

Workshop Debrief

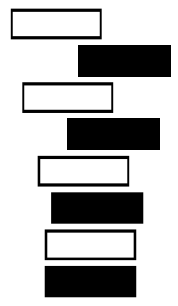

Threads are an effective curricular integration and accountability mechanism

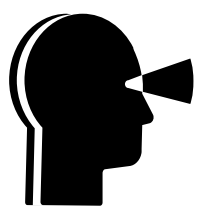

Thread directors can ensure a smooth course to thread transition

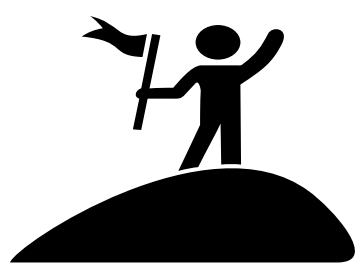

The RAMP framework can be used to develop a longitudinal thread from scratch or from an existing subject-based course

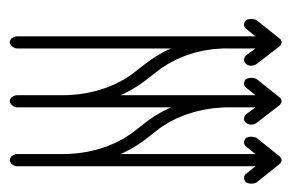

Independent and dependent threads allow for different approaches to integration and instructional delivery

Practice Tips for Thread Directors

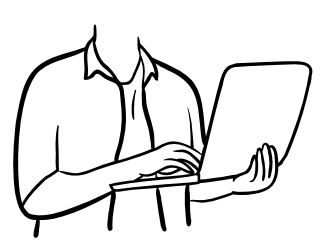

Review all related objectives from the legacy curriculum.

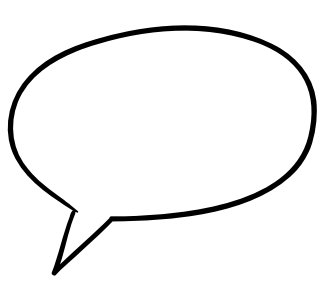

Communicate proactively with course directors.

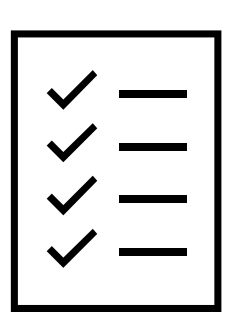

Create a 4-year curricular overview before creating content.

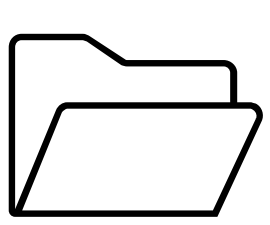

Develop an electronic system to organize and plan thread content.

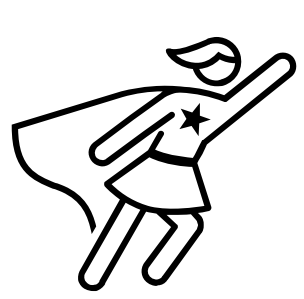

Choose a thread director who is invested in the process.

Depth and breadth of thread of Ethics (Example mapping of MS1 Ethics Thread topics)

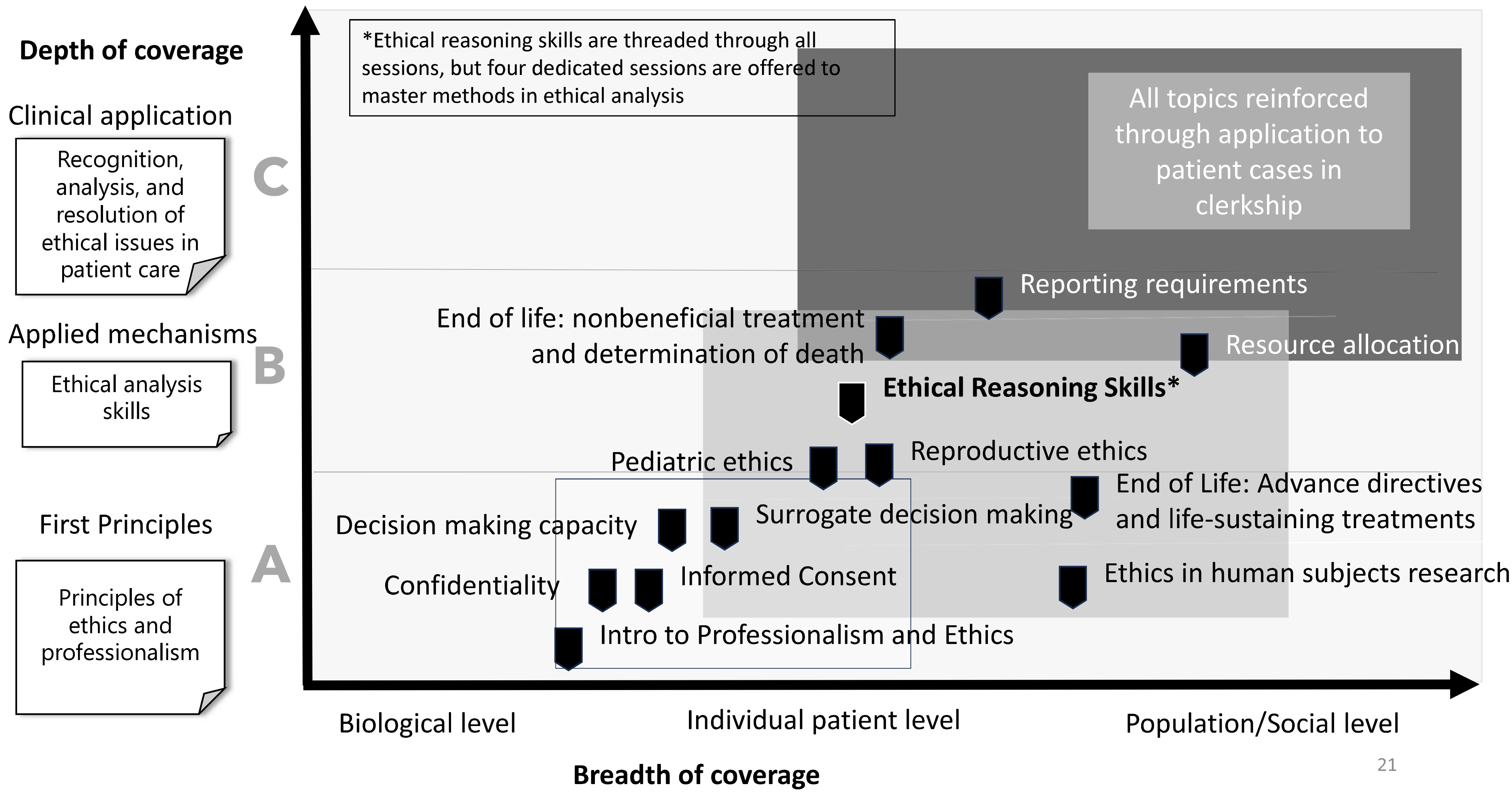

Blank worksheet templates

Objective:
Arrange key thread skills into 3 anchor points

Identify and arrange the skills under their respective anchor points

3 key skills

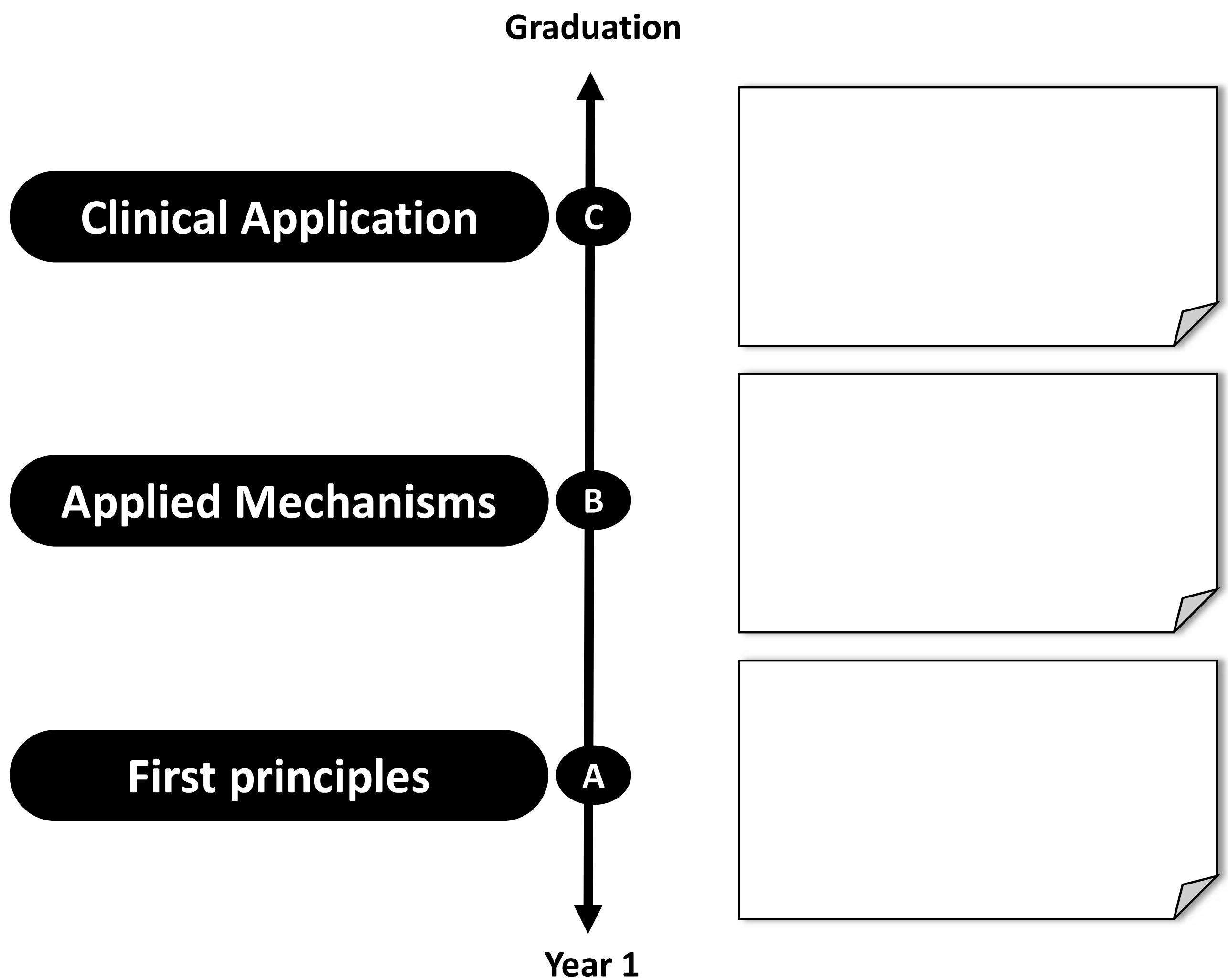

Objective:
Map depth and breadth of thread using anchor points.

Move your anchor points from previous step on y-axis.

Illustrate where anchor points are covered across curriculum. They can stretch across levels.

Foundations of Medicine

Organ systems and Clinical skills

Clerkships

Depth and Breadth of thread

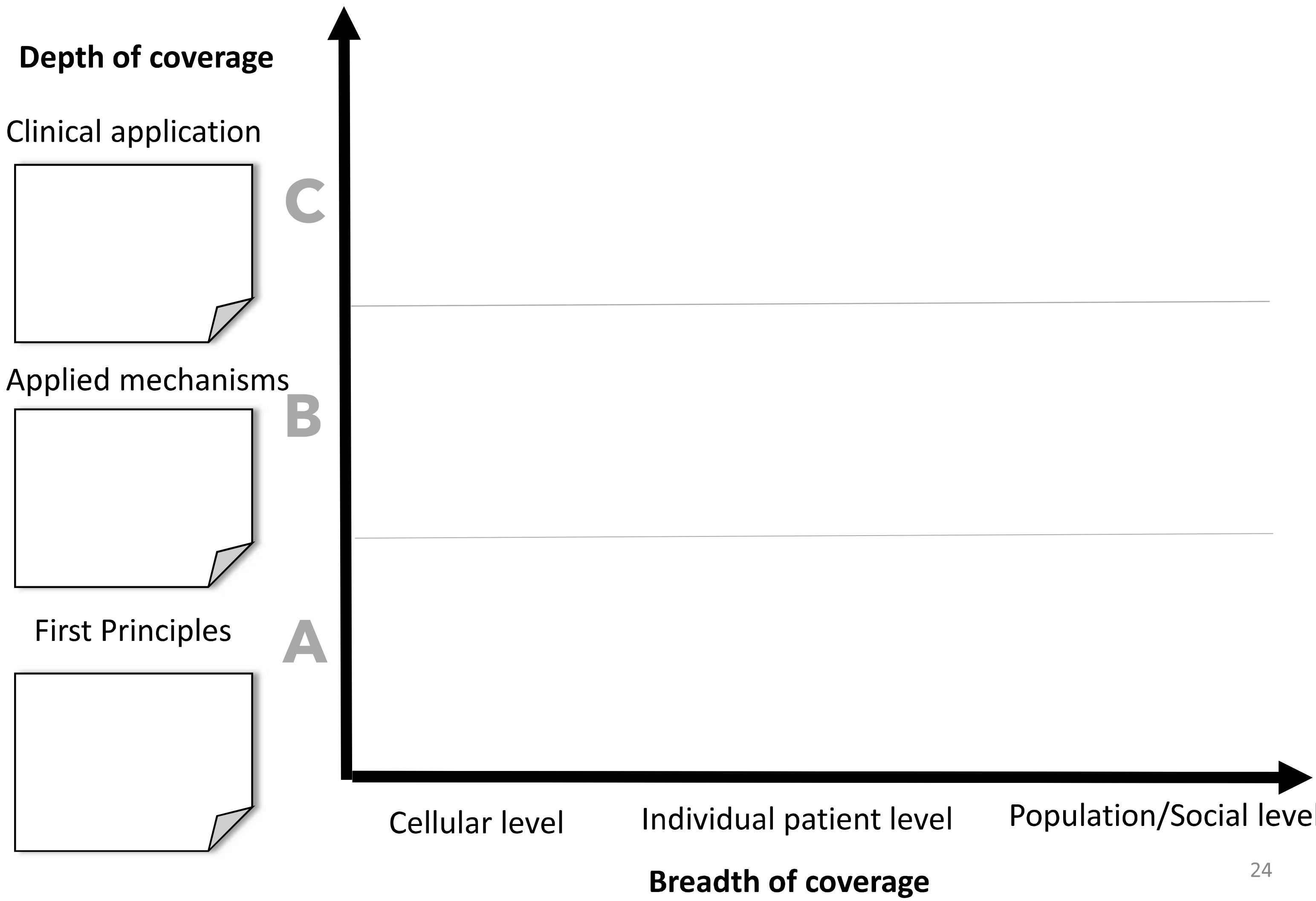

Supplement: Supplementary file 1 [file 40670_2026_2892_MOESM1_ESM.pdf]
